# Non-Invasive Imaging of Facial Erythema Treated with a Transdermal Delivery System

**DOI:** 10.3390/jcm15218332

**Published:** 2026-10-26

**Authors:** Wiktoria Odrzywołek, Dagmara Kuca, Małgorzata Bożek, Anna Deda, Sławomir Wilczyński

**Affiliations:** 1 Department of Basic Biomedical Science, Faculty of Pharmaceutical Sciences in Sosnowiec, Medical University of Silesia, 40-055 Katowice, Poland; d201202@365.sum.edu.pl (D.K.); malgorzata.bozek@sum.edu.pl (M.B.); swilczynski@sum.edu.pl (S.W.); 2 Department of Practical Cosmetology and Skin Diagnostics, Faculty of Pharmaceutical Sciences in Sosnowiec, Medical University of Silesia, 40-055 Katowice, Poland; adeda@sum.edu.pl

**Keywords:** facial erythema, image analysis, GLCM analysis

## Abstract

(1) **Background**: Facial erythema is spatially heterogeneous and may fluctuate considerably, making objective assessment of treatment-related changes challenging. This study aimed to evaluate changes in facial erythema following a hydroporation-based transdermal delivery protocol using complementary instrumental and image-based methods. (2) **Methods**: Twenty-eight women with persistent facial erythema underwent six treatment sessions performed at weekly intervals. Erythema was assessed at baseline, after three treatments, and after completion of the treatment series using a Mexameter and standardized cross-polarized photography followed by Gray-Level Co-occurrence Matrix (GLCM) texture analysis of the green image channel at 45° and 135° orientations. Representative high-frequency ultrasound images were acquired to illustrate fluid deposition within the dermis immediately after the infusion step. (3) **Results**: The median Mexameter erythema index decreased from 383.0 (IQR 362.5–418.5) at baseline to 297.0 (268.5–337.5) after three treatments and 270.0 (247.3–311.3) after six treatments, with significant differences between all time points (Holm-adjusted *p* < 0.001; r = 0.74–0.86). GLCM analysis demonstrated a significant decrease in contrast and an increase in homogeneity at both 45° and 135° orientations compared with baseline (Holm-adjusted *p* < 0.001; r = 0.74–0.83). No significant differences in either GLCM parameter were observed between the third and sixth treatments (*p* > 0.05). (4) **Conclusions**: The hydroporation-based transdermal delivery protocol was associated with a significant reduction in facial erythema. Mexameter and GLCM analysis provided complementary quantitative information on treatment response.

## 1. Introduction

Vascular skin is most commonly observed in the facial region and is characterized by the presence of visible superficial vessels, including telangiectasias and a tendency toward persistent or transient erythema [1,2]. These changes result from the dilation of superficial dermal blood vessels, particularly capillaries and small arterioles, often associated with impaired vascular reactivity and structural alterations within the microcirculation. Telangiectasias may occur as primary lesions related to genetic predisposition or as secondary changes associated with environmental and endogenous factors such as ultraviolet exposure, aging, inflammation, or hormonal influences [3,4]. Neurovascular mechanisms are also involved in the regulation of skin blood flow and their dysregulation may further contribute to vascular instability and erythema.

Various therapeutic strategies are used in the management of vascular skin, including approaches aimed at improving vascular function, reducing inflammation, and strengthening the skin barrier. Depending on the clinical presentation, treatment may include topical therapies, intense pulsed light, and vascular lasers [5,6]. One of the emerging methods involves the transdermal delivery of active substances into the dermis.

Hydroporation-based systems enable the delivery of liquid formulations into the skin using a high-velocity jet stream, which facilitates penetration through the epidermal barrier and allows deposition of substances within the dermis. Histological studies have demonstrated that such systems can achieve a relatively homogeneous distribution of low-viscosity solutions within the dermal compartment, reaching depths exceeding 1 mm, while maintaining minimal disruption of the epidermis under controlled conditions [7].

Regardless of the therapeutic approach used, reliable assessment of treatment outcomes in facial erythema remains difficult because of its heterogeneous and dynamic nature. This creates a need for objective and reproducible methods capable of detecting changes over time [8]. Although visual assessment is widely used because of its simplicity and clinical accessibility, it is subjective and may be influenced by observer interpretation and examination conditions. Objective methods include colorimetric, spectroscopic, reflectance-based, and imaging-based techniques [2,9]. These approaches differ in both their measurement principles and the size of the assessed area: probe-based instruments provide quantitative data from a relatively small, predefined site, whereas imaging methods enable non-contact evaluation of larger regions of interest [10]. This distinction is relevant in facial erythema, which may vary considerably across the facial surface and therefore may not be fully captured by measurements from a single location.

Standardized clinical photography enables quantitative assessment of both the intensity and spatial distribution of facial erythema. Cross-polarized illumination reduces specular reflections and surface glare, thereby improving the visualization of subsurface vascular features and erythema. By enhancing the contrast between erythematous areas and the surrounding skin, this approach may also facilitate more objective characterization of skin color and its changes over time [11]. Image-based analysis also enables quantitative assessment of spatial variations in skin appearance that single-spot measurements may fail to detect [12,13].

Among quantitative image analysis methods, the Gray Level Co-occurrence Matrix (GLCM) has been used for the assessment of skin texture. By analyzing spatial relationships between pixel intensities, GLCM provides quantitative parameters such as contrast and homogeneity, which describe variations in image texture [14,15]. In facial erythema, these parameters may provide information on spatial variations in skin appearance that are not readily captured by visual assessment alone. Subtle changes in skin appearance associated with vascular alterations can be captured through quantitative image analysis, improving the sensitivity of treatment evaluation. In this study, clinical images were further processed by separating individual RGB color channels prior to GLCM analysis, enabling texture analysis within a selected color channel relevant to erythema assessment.

## 2. Materials and Methods

### 2.1. Patients

Twenty-eight females aged 20 to 62 (mean age: 39 ± 12 years) presenting with persistent facial erythema completed a series of six weekly JetPeel PRO (TavTech, Yehud, Israel) procedures. Exclusion criteria included Fitzpatrick skin phototypes IV, V, and VI, systemic isotretinoin therapy within the previous 6 months, dermabrasion or dermal filler treatment within the previous 12 months, chemical peeling within the previous 3 months, skin tanning within the 6 weeks preceding the start of the study, active viral, bacterial, or fungal skin infections, pregnancy or lactation, active malignancy, anticoagulant therapy, vitiligo, tattoos or permanent makeup within the treatment area, botulinum toxin treatment within the previous 6 months, and surgical procedures within the treatment area during the previous 3 months. Participants who declined or withdrew consent were also excluded.

This study was conducted between April and May 2022 in accordance with the principles outlined in the Declaration of Helsinki and was approved by the Ethics Committee of the Medical University of Silesia (approval number: PCN/0022/KB1/12/I/20, issued on 19 May 2020). All participants provided informed, voluntary consent to take part in the research.

### 2.2. Treatments

The transdermal delivery procedure was performed using a hydroporation-based system (JetPeel technology, Yehud, Israel). The system generates a high-velocity jet stream that enables non-contact delivery of liquid formulations into the skin. The jet stream reaches subsonic velocities of approximately 720 km/h. The procedure was performed using dedicated applicators designed for controlled delivery of the jet stream.

A total of six treatment sessions were performed at week intervals. Each treatment session consisted of three consecutive stages: lymphatic drainage, exfoliation, and infusion.

The procedure was initiated with a lymphatic drainage phase performed using a JetCare Hydro solution at an approximate distance of 7 cm from the skin surface. At this distance, the jet stream induces gentle deformation of the skin surface and when combined with controlled draining movements, supports superficial lymphatic flow.

This was followed by an exfoliation step, during which 4 mL of Mandelic & Salicylic solution (4% mandelic acid, 2% salicylic acid) was delivered. The jet stream was applied initially at a 90° angle relative to the skin surface and subsequently at a 45° angle. This approach allowed the kinetic energy of the jet stream to remove loosely attached corneocytes and surface impurities. A relatively low fluid flow rate was maintained to minimize the formation of a boundary layer on the skin surface, which could otherwise reduce the efficiency of particle removal and limit penetration of the jet stream. The distance between the device tip and the skin surface during this stage was maintained at approximately 8 mm.

Following exfoliation, the infusion phase was performed using 4 mL of Soothing solution, with the jet stream applied perpendicularly to the skin surface (90° angle) and at a distance of approximately 8 mm. Under these conditions, deformation of the skin induced by the jet stream facilitates the formation of transient microchannels, enabling transdermal delivery of active substances into the dermis (barophoresis effect). Subsequently, an additional 1 mL of Glow Booster formulation was applied to the erythema-affected areas of the cheeks in all participants (Supplementary File S1).

### 2.3. Ultrasound Imaging

**Figure 1:**
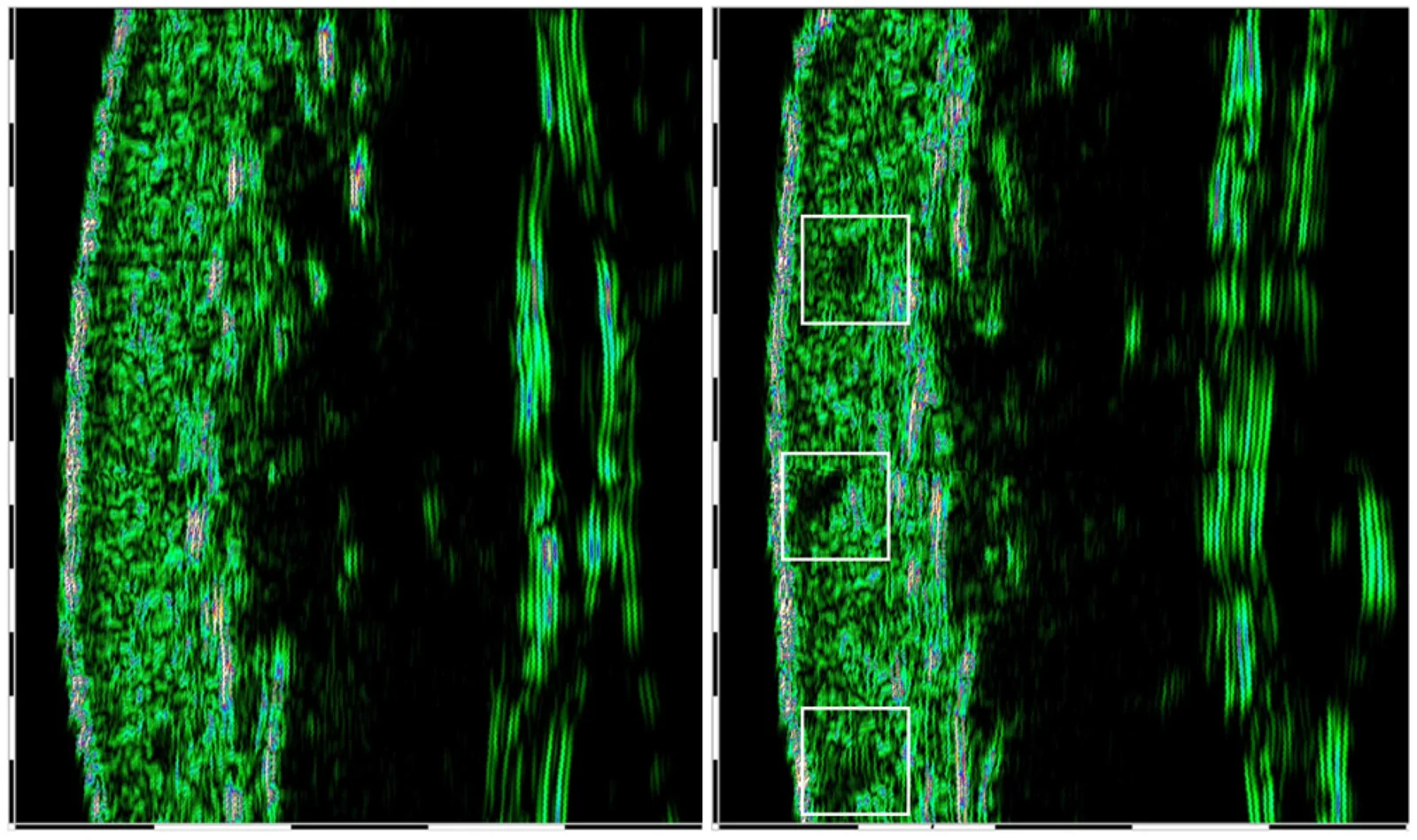
Representative high-frequency ultrasound images of the skin acquired before (**left**) and immediately after (**right**) the test application of the transdermal infusion procedure. The fluid was delivered sequentially at multiple locations using a single-outlet handpiece, with a total application time of approximately 10 s per 1 cm^2^. White rectangles indicate regions of fluid distribution within the dermal compartment following the procedure.

**Figure 2:**
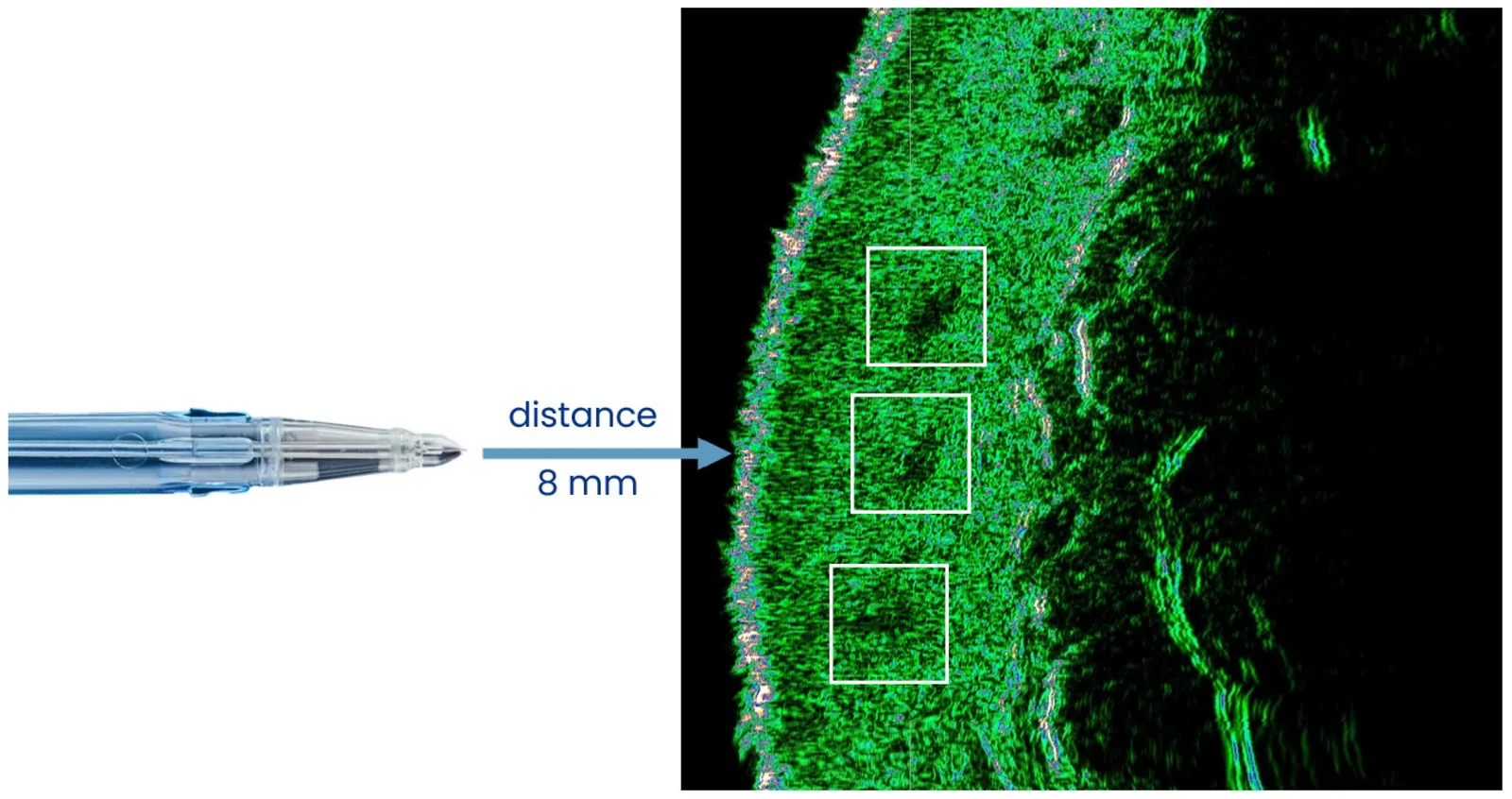
Schematic representation of the transdermal infusion procedure and corresponding high-frequency ultrasound image of the skin. The single-outlet applicator generated one jet stream and was positioned approximately 8 mm from the skin surface, perpendicular to the treatment area. The fluid was applied sequentially at multiple locations within the examined area, with a total application time of approximately 10 s per 1 cm^2^. White rectangles indicate regions of fluid distribution within the dermal compartment following the procedure.

High-frequency ultrasonography was performed to qualitatively visualize the distribution of the infused solution within the dermis following the transdermal delivery procedure (Appendix A). High-frequency ultrasonography (HFUS) was performed using the DUB SkinScanner (tpm-taberna pro medicum, Germany) operating in B-scan mode with a 33 and 50 MHz transducers. HFUS was used for illustrative purposes only and was not included in the outcome assessment (Figure 1 and Figure 2).

### 2.4. Clinical Photographs

Clinical photographs were acquired using the FotoMedicus medical photography system (ELFO, Łódź, Poland), equipped with ELFO MIQRO PRO flash units with a maximum flash energy of 250 Ws. According to the manufacturer’s technical specifications for the MIQRO PRO series, the flash units have a nominal color temperature of 5400 ± 200 K. The flash energy was set to 2.5 on the manufacturer’s relative adjustment scale, and this setting remained unchanged throughout all examination sessions.

**Figure 3:**
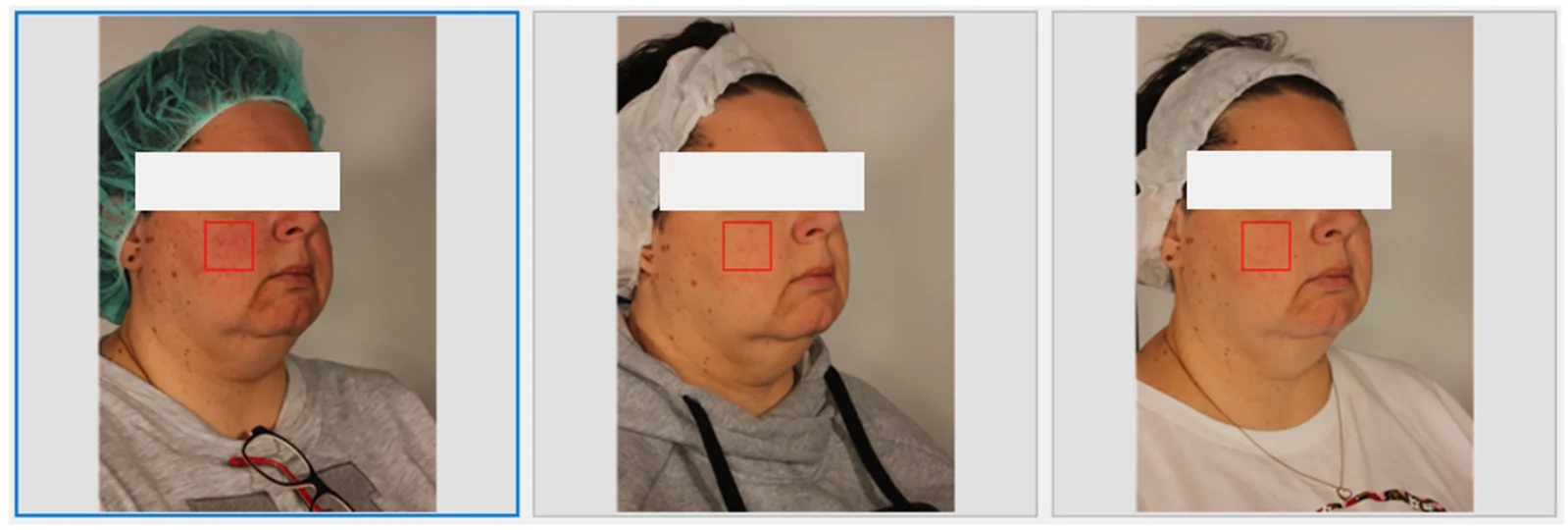
Representative standardized cross-polarized photographs obtained at baseline (**left**), after three treatments (**center**), and after completion of six treatments (**right**). Red rectangles indicate the region of interest used for quantitative image texture analysis.

The system utilizes a reference overlay (“ghost image”) that allows precise repositioning of the subject during subsequent imaging sessions, improving consistency of image alignment between baseline and follow-up photographs. Standardized illumination parameters, including fixed flash intensity and stable color temperature, were applied to ensure consistent rendering of skin color and structural details. Photographs were acquired at a fixed camera-to-subject distance of 80 cm, with reproducible positioning ensured by a dedicated positioning mat and the reference overlay function. The images had a resolution of 3464 × 5202 pixels. All images were captured using cross-polarized light to reduce surface glare and enhance visualization of deeper skin features (Figure 3).

Photographs were acquired at three assessment time points:

- before the treatment series,
- one week after the third treatment,
- one week after the sixth treatment.

Prior to image acquisition, all participants underwent a 30 min acclimatization period in the examination room to minimize the influence of environmental factors on skin appearance. The ambient temperature was maintained at 22 ± 1 °C, with a relative humidity of 40–55%, to ensure thermal comfort and minimize the potential influence of environmental factors on cutaneous microcirculation, skin appearance, and erythema intensity. Additionally, participants were instructed to avoid activities and external stimuli that could induce erythema, such as physical exertion, exposure to extreme temperatures, and the use of irritating cosmetic products, before imaging.

For quantitative image analysis, a standardized region of interest (ROI) measuring 500 × 500 pixels was used for all photographs. The ROI was automatically positioned at the same anatomical location for each participant across all three assessment time points (baseline, after three treatments, and after six treatments). This procedure ensured consistent ROI dimensions and positioning, minimizing variability associated with differences in the analyzed image area.

Texture analysis was performed using the Gray-Level Co-occurrence Matrix (GLCM), a statistical method used to describe image texture by evaluating spatial relationships between pixel intensity values. The GLCM quantifies how often a pixel with a specific intensity occurs in a predefined spatial relationship to another pixel with a different or similar intensity, enabling extraction of textural features such as contrast and homogeneity.

Spatial relationships in the GLCM are defined by direction and distance between pixel pairs. Commonly used orientations include 0°, 45°, 90°, and 135°. In the present study, GLCM parameters were analyzed at 45° and 135° orientations using a pixel distance of one.

**Figure 4:**
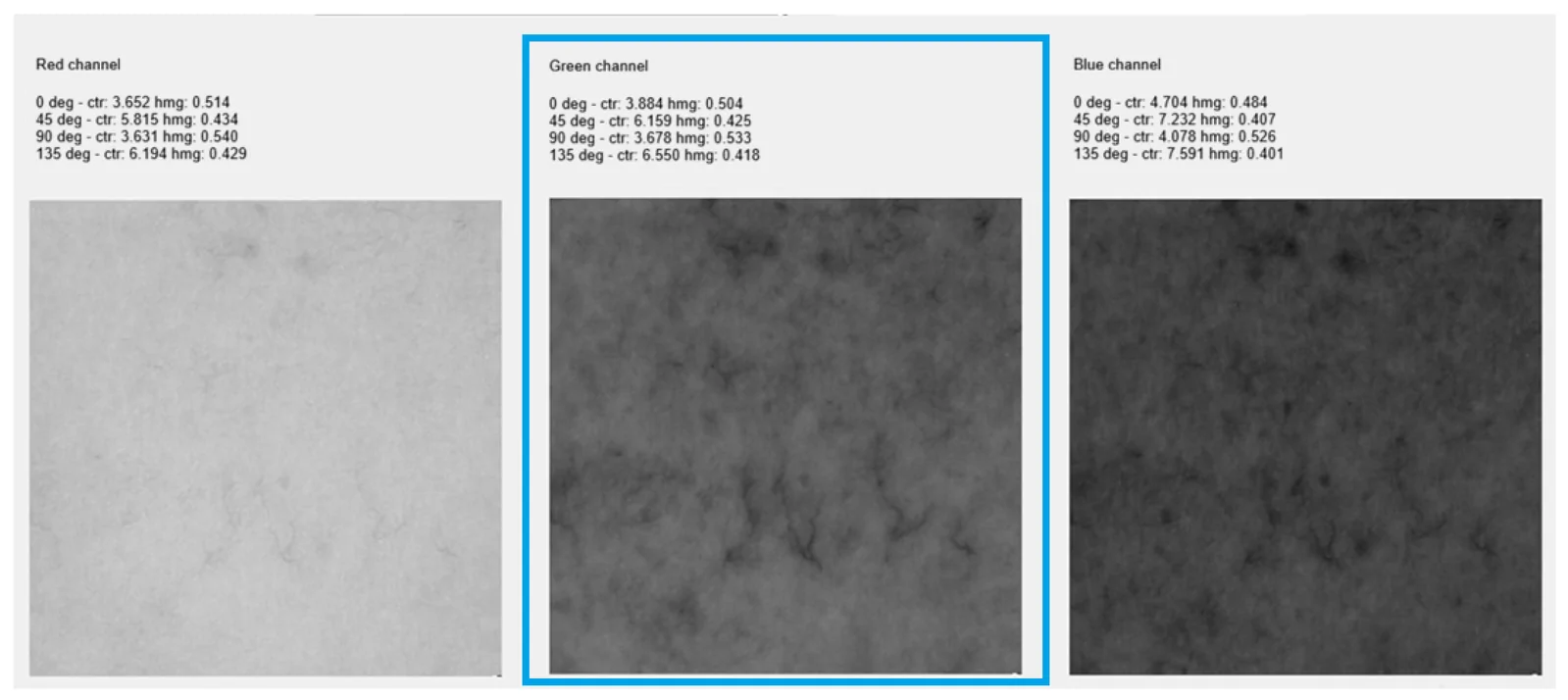
Representative decomposition of the selected region of interest into red, green, and blue (RGB) image channels for texture analysis. The green channel (center) was selected a priori for subsequent GLCM analysis based on previous evidence supporting its utility in the assessment of erythema and vascular skin changes.

Prior to texture extraction, images were decomposed into individual RGB color channels (red, green, and blue). For texture analysis in the present study, the green channel was selected based on previous evidence supporting its utility in image-based assessment of erythema and vascular skin changes (Figure 4) [16].

GLCM calculations were performed using the Accord. Imaging library (Accord.NET Framework), which enables efficient and reproducible computation of texture parameters.

Two texture features were analyzed: contrast and homogeneity. Contrast measures the degree of local intensity variation between neighboring pixels and reflects structural heterogeneity of the image. Higher contrast values indicate greater variability in pixel intensities and correspond to more irregular textures, whereas lower values indicate smoother and more uniform structures. In highly uniform images, contrast approaches zero.

Homogeneity describes the similarity of pixel intensities within the image and reflects the uniformity of the analyzed structure. Its values range from 0 to 1, with higher values indicating greater uniformity. Homogeneity reaches a value of 1 when all pixels have identical intensity.

### 2.5. Erythema Assessment

Erythema intensity was evaluated using a Mexameter^®^ (MX18, Courage-Khazaka Electronics GmbH, Cologne, Germany) device, which enables non-invasive quantification of skin chromophores based on light absorption and reflection. The probe emits light at specific wavelengths, including 568 nm (green) and 660 nm (red), which are used for erythema assessment.

The measurement is based on differential absorption of light by hemoglobin. One of the wavelengths corresponds to the absorption peak of hemoglobin, while the second is selected to minimize the influence of other skin chromophores, allowing for more specific evaluation of vascular-related color changes. The obtained values are expressed as an erythema index on an arbitrary scale ranging from 0 to 999 (Supplementary File S2).

Measurements were performed using a probe with a diameter of approximately 5 mm. Mexameter measurements were obtained at the same three assessment time points as the clinical photographs: before the treatment series, one week after the third treatment, and one week after the sixth treatment. To ensure the reproducibility of Mexameter measurements, the measurement sites were marked on clinical photographs, which served as references for identifying the same anatomical locations at each subsequent examination. All measurements were conducted under standardized environmental conditions.

### 2.6. Statistical Analysis

Statistical analysis was performed using Statistica 13.3 (TIBCO Software Inc. (2017). Statistica (data analysis software system), version 13, Palo Alto, CA, USA) and Python (SciPy 1.18, statsmodels 0.15). The normality of data distribution was assessed using the Shapiro–Wilk test. As the assumption of normality was not met for all variables, non-parametric methods were applied, and data are presented as median and interquartile range (Q1–Q3). Differences between the three assessment time points were analyzed using the Friedman test, with Kendall’s coefficient of concordance (W) reported as a measure of effect size. When a significant overall difference was detected, pairwise post hoc comparisons were performed using the Wilcoxon signed-rank test. To control the family-wise type I error rate, the *p*-values of all 15 post hoc comparisons (three pairwise comparisons for each of the five outcome variables) were adjusted jointly using the Holm–Bonferroni method. Effect sizes for pairwise comparisons were expressed as r, calculated from the standardized Wilcoxon test statistic and the number of participants (*N* = 28), and interpreted as small (0.1), medium (0.3), or large (0.5). The magnitude of change was additionally quantified using the Hodges–Lehmann estimator of the median paired difference with its 95% confidence interval. A two-sided *p*-value < 0.05 (after adjustment, where applicable) was considered statistically significant.

## 3. Results

**Figure 5:**
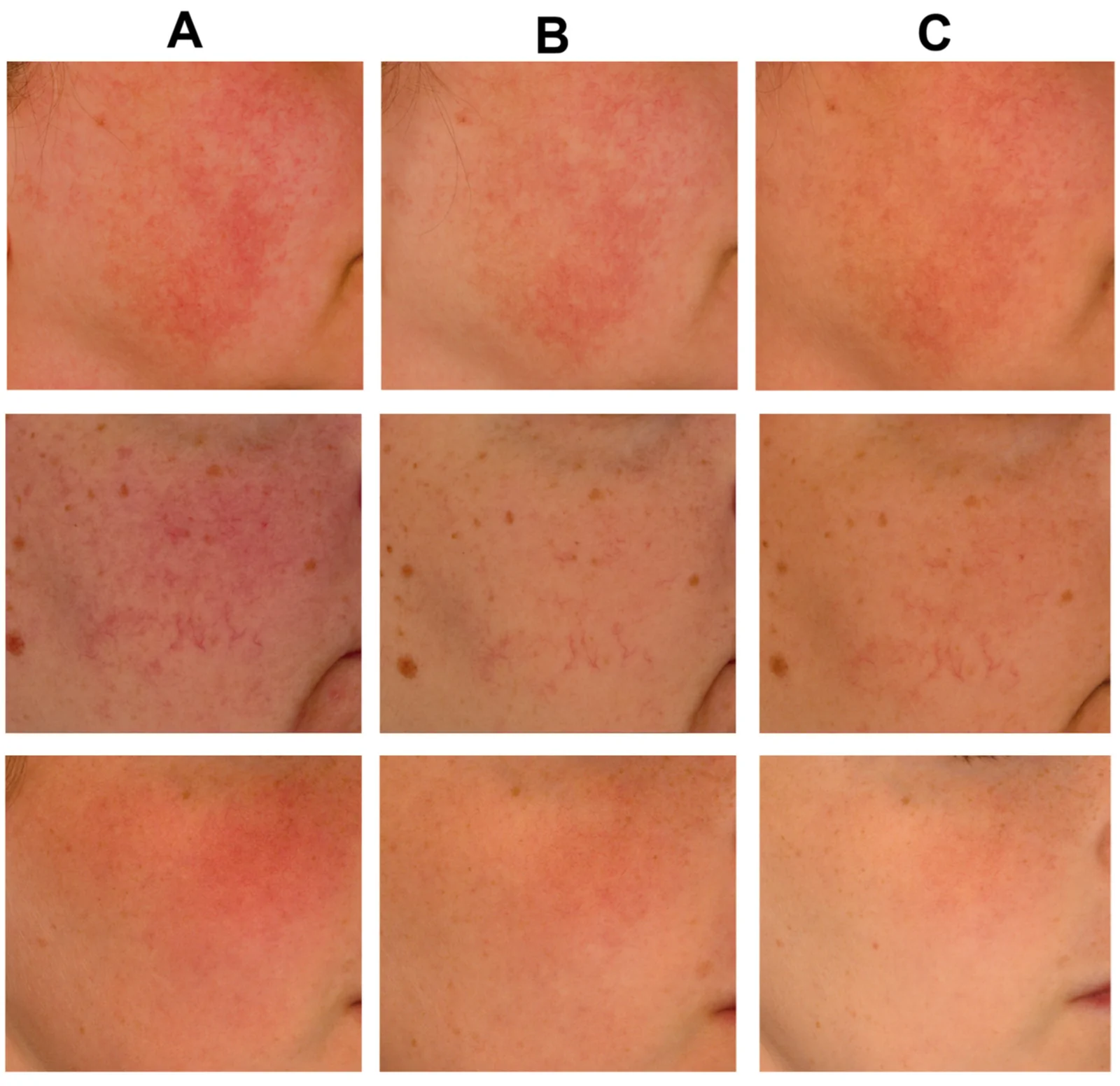
Standardized cross-polarized photographs of three participants (rows) at baseline (**A**), after three treatments (**B**), and after completion of six treatments (**C**), illustrating the range of clinical response to treatment.

All participants completed the treatment series, and no adverse events were observed during the study. Representative photographs illustrating the range of clinical response observed across participants are shown in Figure 5.

**Figure 6:**
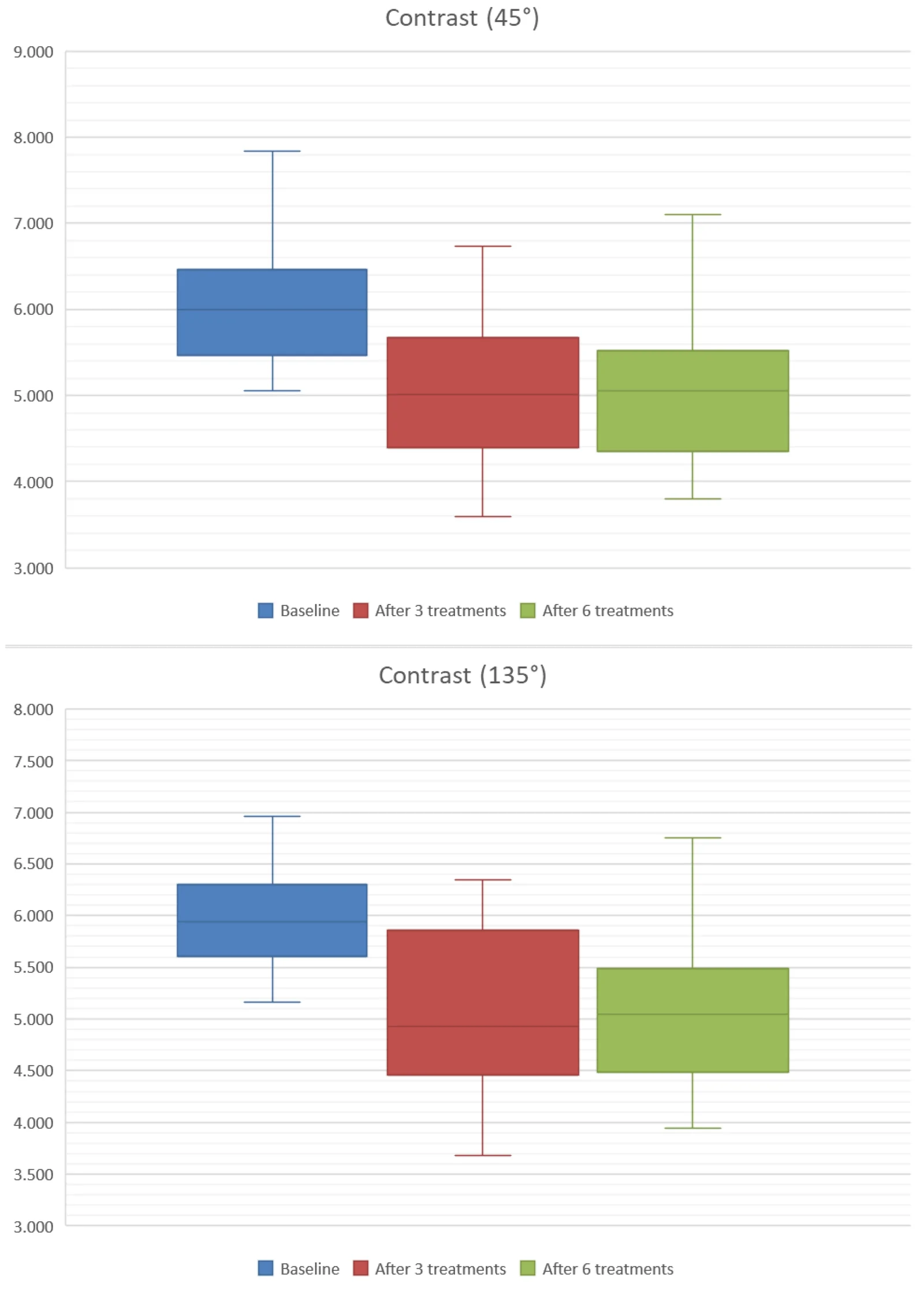
Changes in GLCM contrast in the green image channel at baseline, after three treatments, and after six treatments, analyzed at 45° and 135° orientations. Boxes represent the interquartile range (Q1–Q3), the central line indicates the median, and whiskers indicate the minimum and maximum values. Brackets indicate statistically significant pairwise comparisons (Wilcoxon signed-rank test, Holm-adjusted *p*-values; see Table 2).

Changes in image texture were evaluated using GLCM-derived contrast and homogeneity parameters at 45° and 135° orientations. Contrast decreased significantly from baseline at both analyzed orientations. At 45°, the median contrast decreased from 5.999 at baseline to 5.017 after three treatments and 5.053 after six treatments. At 135°, the corresponding median values were 5.939, 4.927, and 5.044, respectively (Table 1). For both orientations, the overall differences between time points were statistically significant (Friedman test, *p* < 0.001; Kendall’s W = 0.51 and 0.46, respectively). Analysis showed significant reductions in contrast after three and six treatments compared with baseline (Holm-adjusted *p* < 0.001 for all comparisons; r = 0.74–0.80; Table 2), whereas no significant difference was observed between the third and sixth treatments at either orientation (unadjusted *p* ≥ 0.716; adjusted *p* = 1.000) (Figure 6). The small difference in median contrast at 45°between the third and sixth treatments (5.017 vs. 5.053) was not statistically significant and showed no consistent direction (an increase in 16 and a decrease in 12 participants; Hodges–Lehmann median difference 0.050, 95% CI −0.267 to 0.361).

**Figure 7:**
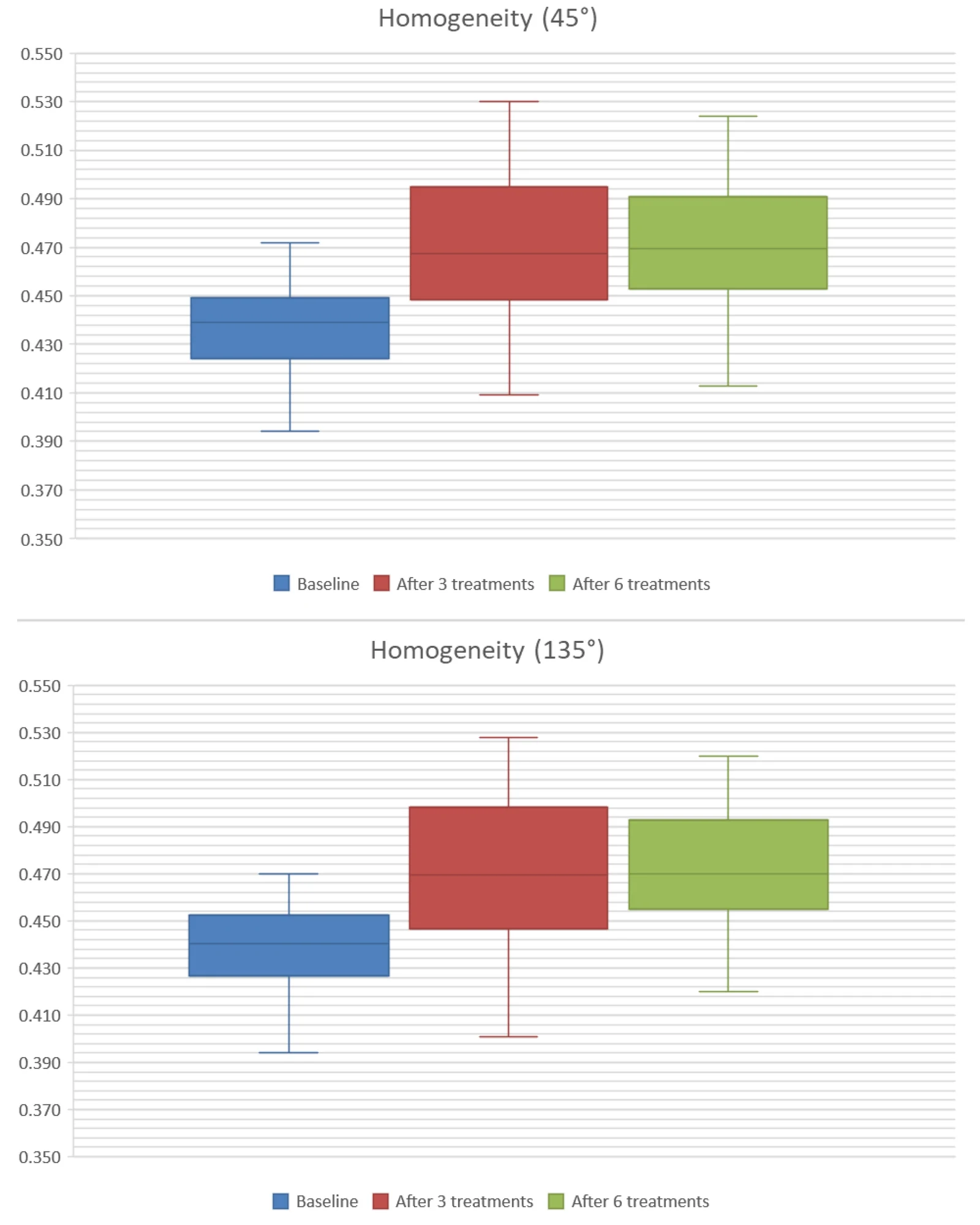
Changes in green-channel GLCM homogeneity at 45° and 135° orientations across the three assessment time points. Boxes represent the interquartile range (Q1–Q3), the central line indicates the median, and whiskers indicate the minimum and maximum values. Brackets indicate statistically significant pairwise comparisons (Wilcoxon signed-rank test, Holm-adjusted *p*-values; see Table 2).

Homogeneity increased significantly from baseline at both analyzed orientations. At 45°, the median homogeneity increased from 0.439 at baseline to 0.468 after three treatments and 0.470 after six treatments. At 135°, the corresponding median values were 0.441, 0.470, and 0.470, respectively (Table 1). For both orientations, the overall differences between time points were statistically significant (Friedman test, *p* < 0.001; Kendall’s W = 0.51 and 0.53, respectively). Analysis showed significant increases in homogeneity after three and six treatments compared with baseline (Holm-adjusted *p* < 0.001 for all comparisons; r = 0.79–0.83; Table 2), whereas no significant difference was observed between the third and sixth treatments at either orientation (unadjusted *p* ≥ 0.838; adjusted *p* = 1.000) (Figure 7).

**Figure 8:**
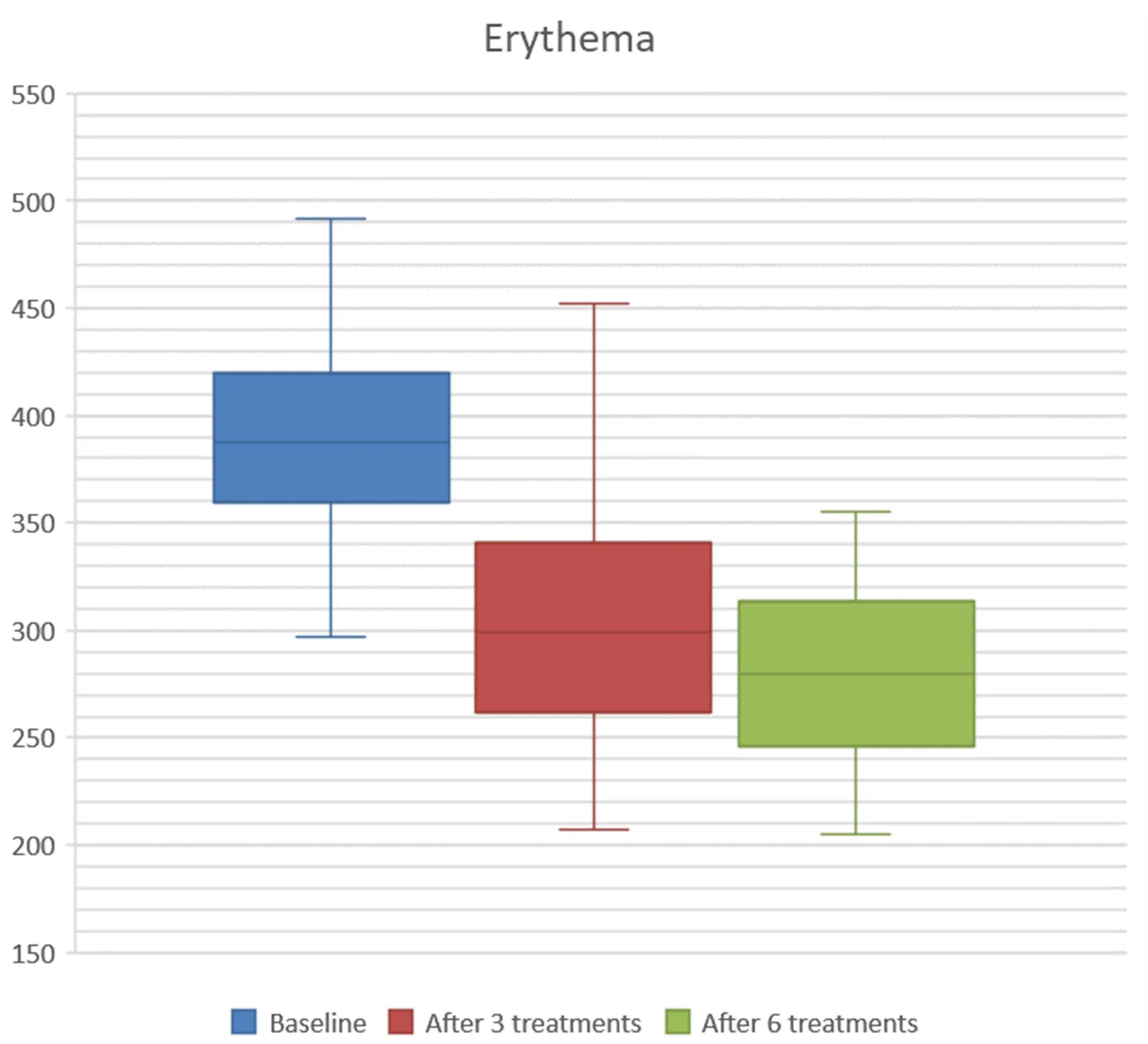
Changes in the Mexameter erythema index at baseline, after three treatments, and after six treatments. Boxes represent the interquartile range (Q1–Q3), the central line indicates the median, and whiskers indicate the minimum and maximum values. Brackets indicate statistically significant pairwise comparisons (Wilcoxon signed-rank test, Holm-adjusted *p*-values; see Table 2).

The median erythema index decreased from 383.0 (IQR 362.5–418.5) at baseline to 297.0 (268.5–337.5) after three treatments and further to 270.0 (247.3–311.3) after six treatments (Table 1). The overall difference between time points was statistically sig-nificant (Friedman test, χ^2^(2) = 41.36, *p* < 0.001; Kendall’s W = 0.74). Analysis showed significant differences between all assessment time points (Holm-adjusted *p* < 0.001 for all comparisons; r = 0.74–0.86; Table 2) (Figure 8).

## 4. Discussion

Objective evaluation of persistent facial erythema remains difficult due to its dynamic and heterogeneous nature. Despite the availability of various therapeutic approaches, assessment of treatment outcomes is complicated by fluctuations in erythema associated with environmental conditions, emotional stimuli, physical activity, or temperature [17]. Furthermore, commonly used clinical grading scales remain valuable in routine practice and clinical trials, but their ordinal and observer-dependent nature may limit their ability to detect changes in erythema severity over time [18,19]. Consequently, there is an increasing need for quantitative, reproducible methods capable of evaluating treatment-induced changes from different perspectives [20,21,22]. In the present study, we combined instrumental erythema assessment with standardized cross-polarized photography and quantitative texture analysis to provide a more comprehensive evaluation of treatment response.

Significant changes in image texture were observed following treatment. GLCM contrast decreased, whereas homogeneity increased compared with baseline, with a consistent direction of change at both 45° and 135° orientations. These parameters describe different aspects of spatial intensity distribution within an image: lower contrast indicates reduced local variation between neighboring pixels, whereas higher homogeneity reflects greater similarity of their intensities. In the context of facial erythema, the simultaneous decrease in contrast and increase in homogeneity may therefore indicate a more uniform appearance of the analyzed skin area.

This direction of change is consistent with recent studies using GLCM analysis to evaluate vascular and erythematous facial skin. Lipka-Trawińska et al. applied GLCM analysis to standardized cross-polarized photographs of facial vascular lesions following IPL treatment and reported a significant reduction in contrast together with an increase in image homogeneity [16]. Similarly, Deda et al., in a study co-authored by some of the present authors, observed an approximately 17% reduction in GLCM contrast and a 4–5% increase in homogeneity following polychromatic light treatment in patients with rosacea. Moreover, GLCM parameters showed strong correlations with Clinician Erythema Assessment scores, supporting their relevance for quantitative evaluation of visible erythematous changes [23]. Although these studies investigated light-based vascular treatments and therefore differ substantially from the treatment protocol used in the present study, the comparable direction of GLCM changes supports the interpretation of decreasing contrast and increasing homogeneity as quantitative indicators of a more uniform appearance of erythematous skin. The magnitude of the changes observed in the present study (a decrease of approximately 15–17% in median contrast and an increase of approximately 7% in median homogeneity relative to baseline) was comparable to that reported by Deda et al.

The use of image-based analysis may provide information that is not fully captured by a single erythema index. Tao et al. demonstrated that image-derived measures of both erythema intensity and affected facial area were associated with clinical severity in rosacea [21]. Similarly, Logger et al. emphasized that digital photographs allow assessment of both larger and smaller erythematous areas and enable retrospective analysis of treatment-related changes [20]. These observations are particularly relevant to the present study because GLCM does not quantify erythema intensity directly; instead, it characterizes spatial relationships between pixel intensities within a defined region. Texture analysis may therefore complement localized instrumental measurements by providing information on how uniformly erythema is distributed across the analyzed skin area.

For texture analysis in the present study, the green channel of the RGB images was selected a priori, based on previous image-based studies of vascular skin. Notably, in a previous study involving members of the present research team Lipka-Trawińska et al. analyzed GLCM parameters separately across RGB channels and found that treatment-related changes in vascular skin appearance were particularly pronounced in the green channel [16]. The use of green-channel information is also consistent with image-based approaches to erythema assessment in which differences between red and green image components have been exploited to quantify redness [20]. Because GLCM parameters describe image texture rather than redness, a contribution of surface-related changes to the observed results cannot be excluded, although the exfoliation step used low acid concentrations and cross-polarized imaging attenuates the influence of surface microrelief. The GLCM findings should therefore be interpreted as reflecting a more uniform overall appearance of the analyzed skin area.

The Mexameter and GLCM parameters showed different patterns of change over the treatment course. While Mexameter measurements demonstrated a progressive reduction in erythema across consecutive assessments, GLCM contrast and homogeneity changed significantly during the first half of the treatment series and subsequently remained relatively stable. This difference may partly reflect fundamental differences in the spatial scale and type of information provided by the two methods. The Mexameter generates a localized erythema index from a small skin area of approximately 5 mm in diameter, whereas image-based analysis integrates information across a substantially larger region of interest. This distinction is particularly relevant in facial erythema, which is often spatially heterogeneous and may include areas of diffuse redness, visible telangiectasias, normally colored skin, and pigmentation within the same anatomical region. Saknite et al. directly compared single-spot Mexameter measurements with RGB-based erythema imaging in patients with erythematotelangiectatic rosacea and showed that image-derived erythema indices were more strongly associated with clinical erythema grading than Mexameter measurements [24]. These observations suggest that localized instrumental measurements and image-based analyses may provide complementary information by capturing different aspects of treatment-related changes in facial erythema.

High-frequency ultrasonography was used only to illustrate the infusion step. The ultrasonographic appearance immediately after the procedure was compatible with fluid deposition within the dermis, which is the expected finding after high-velocity jet application and is consistent with previous histological observations [7]. However, HFUS does not allow identification of the administered substances or determination of their concentration within the tissue, and the images were not related to the erythema outcome; they therefore do not provide evidence that specific active ingredients reached the dermis or that their delivery contributed to the observed reduction in erythema.

Several components of the formulations administered during the present treatment protocol have previously been investigated in the management of erythematous, rosacea-prone, and sensitive skin. Potassium azeloyl diglycinate appears particularly relevant, as a randomized, double-blind, placebo-controlled study demonstrated that a topical formulation containing 5% potassium azeloyl diglycinate significantly reduced facial erythema assessed both instrumentally with the Mexameter and by clinical evaluation in patients with stage I–II rosacea [25]. Niacinamide has likewise been associated with improvement of rosacea-prone skin, primarily through restoration of epidermal barrier function and increased stratum corneum hydration, accompanied by improvement in clinical signs and symptoms [26]. More recently, troxerutin-containing topical formulations have been reported to improve skin hydration and barrier integrity while reducing regional facial erythema, particularly within the cheeks and nose [27]. Although these observations provide biological plausibility for the use of such ingredients in erythematous skin, the present study evaluated a multimodal protocol comprising drainage, exfoliation, and transdermal infusion with several multi-component formulations. Consequently, the observed clinical improvement should be attributed to the overall treatment protocol rather than to the effects of any individual active ingredient.

### Limitations

- The study was a single-arm, non-randomized, unblinded study without an untreated or vehicle-treated control group. Therefore, regression to the mean, seasonal variation (the study was conducted in spring, during increasing UV exposure), and expectation effects cannot be excluded as alternative explanations for the observed changes.
- No standardized clinical scales were used to assess erythema severity.
- HFUS imaging was used only for qualitative visualization, without quantitative analysis.
- The study did not include clinician- or patient-rated assessment of erythema; therefore, it cannot be determined whether the observed changes in GLCM parameters are visually perceptible.
- The 45° and 135° orientations are derived from the same image region and are strongly correlated (Spearman’s ρ = 0.75–0.93 across time points); they therefore describe the same texture from two directions and should not be regarded as independent measurements.

## 5. Conclusions

The investigated hydroporation-based protocol resulted in a significant, progressive reduction in facial erythema, as confirmed by Mexameter measurements throughout the treatment series. GLCM analysis showed decreased contrast and increased homogeneity, indicating a more uniform appearance of the erythematous skin area following treatment. Notably, Mexameter and image-based parameters showed distinct temporal patterns of change, suggesting that these methods provide complementary rather than redundant information on treatment response. These findings support further investigation of hydroporation-based transdermal delivery in the management of facial erythema and demonstrate the usefulness of quantitative image analysis for objective treatment assessment.

## Figures and Tables

**Table 1:** Erythema index and green-channel GLCM parameters at the three assessment time points (N = 28). Data are median (Q1–Q3).

Variable	Baseline	After 3 Treatments	After 6 Treatments	Friedman χ^2^(2)	*p*	Kendall’s W
Erythema index (Mexameter)	383.0 (362.5–418.5)	297.0 (268.5–337.5)	270.0 (247.3–311.3)	41.36	<0.001	0.74
Contrast 45°	5.999 (5.496–6.444)	5.017 (4.435–5.667)	5.053 (4.400–5.513)	28.79	<0.001	0.51
Contrast 135°	5.939 (5.619–6.292)	4.927 (4.532–5.815)	5.044 (4.504–5.476)	26.00	<0.001	0.46
Homogeneity 45°	0.439 (0.426–0.449)	0.468 (0.452–0.495)	0.470 (0.453–0.491)	28.50	<0.001	0.51
Homogeneity 135°	0.441 (0.428–0.451)	0.470 (0.448–0.497)	0.470 (0.455–0.491)	29.86	<0.001	0.53

Data are presented as median (Q1–Q3). Effect size for the Friedman test is expressed as Kendall’s coefficient of concordance (W).

**Table 2:** Post hoc pairwise comparisons (Wilcoxon signed-rank test). *p*_adj_: Holm-Bonferroni adjustment across all 15 comparisons. HL Δ: Hodges–Lehmann estimate of the median paired difference (later minus earlier time point) with 95% CI.

Variable	Comparison	Z	*p*	*p*_adj_	r	HL Δ (95% CI)
Erythema index	Baseline vs. 3	3.94	<0.001	<0.001	0.74	−80.8 (−102.0 to −58.5)
	Baseline vs. 6	4.56	<0.001	<0.001	0.86	−106.5 (−126.0 to −83.0)
	3 vs. 6	4.10	<0.001	<0.001	0.77	−16.0 (−34.0 to −6.5)
Contrast 45°	Baseline vs. 3	4.26	<0.001	<0.001	0.80	−1.015 (−1.391 to −0.643)
	Baseline vs. 6	3.94	<0.001	<0.001	0.74	−1.059 (−1.317 to −0.840)
	3 vs. 6	0.36	0.716	1.000	0.07	0.050 (−0.267 to 0.361)
Contrast 135°	Baseline vs. 3	4.21	<0.001	<0.001	0.80	−0.939 (−1.268 to −0.569)
	Baseline vs. 6	4.21	<0.001	<0.001	0.80	−1.020 (−1.263 to −0.763)
	3 vs. 6	0.21	0.838	1.000	0.04	0.038 (−0.314 to 0.308)
Homogeneity 45°	Baseline vs. 3	4.38	<0.001	<0.001	0.83	0.033 (0.020 to 0.047)
	Baseline vs. 6	4.31	<0.001	<0.001	0.81	0.034 (0.027 to 0.044)
	3 vs. 6	0.15	0.882	1.000	0.03	−0.001 (−0.012 to 0.010)
Homogeneity 135°	Baseline vs. 3	4.18	<0.001	<0.001	0.79	0.031 (0.018 to 0.046)
	Baseline vs. 6	4.34	<0.001	<0.001	0.82	0.035 (0.028 to 0.045)
	3 vs. 6	0.21	0.838	1.000	0.04	0.001 (−0.011 to 0.013)

Baseline, 3, and 6 denote baseline, after three treatments, and after six treatments, respectively. *p*, unadjusted *p*-value; *p*_adj_, *p*-value adjusted using the Holm-Bonferroni method across all 15 pairwise comparisons; r = |Z|/√N; HL Δ, Hodges–Lehmann estimate of the median paired difference (later minus earlier time point) with 95% confidence interval.

## Data Availability

Data is contained within the article or Supplementary Materials.

## Supplementary Materials

The following supporting information can be downloaded at: https://www.mdpi.com/article/10.3390/jcm15218332/s1, Supplementary File S1: Ingredients of the formulations used in the treatment protocol; Supplementary File S2: Technical brochure for the Mexameter® MX 18.

## Abbreviations

The following abbreviations are used in this manuscript:

GLCM	Gray-Level Co-occurrence Matrix
HFUS	High-Frequency Ultrasonography
RGB	Red, Green, and Blue

## Appendix A

1. Figure 1. Representative high-frequency ultrasound images of the skin acquired before (left) and immediately after (right) the transdermal infusion procedure.
2. Figure 2. Schematic representation of the transdermal infusion procedure and corresponding high-frequency ultrasound image of the skin.
3. Figure 3. Representative standardized cross-polarized photographs obtained at baseline (left), after three treatments (center), and after completion of six treatments (right).
4. Figure 4. Representative decomposition of the selected region of interest into red, green, and blue (RGB) image channels for texture analysis.
5. Figure 5. Standardized cross-polarized photographs of three participants (rows) at baseline (A), after three treatments (B), and after completion of six treatments (C), illustrating the range of clinical response to treatment.
6. Figure 6. Changes in GLCM contrast in the green image channel at baseline, after three treatments, and after six treatments, analyzed at 45° and 135° orientations.
7. Figure 7. Changes in green-channel GLCM homogeneity at 45° and 135° orientations across the three assessment time points.
8. Figure 8. Changes in the Mexameter erythema index at baseline, after three treatments, and after six treatments.
