# Supplementary material for: Non-Invasive Imaging of Facial Erythema Treated with a Transdermal Delivery System
Source: J Clin Med. 2026 Oct 26;15(21):8332. doi: 10.3390/jcm15218332 (PMC13764176; doi:10.3390/jcm15218332)
Supplement: Supplementary file 1 [file jcm-15-08332-s001.zip › jcm-4576705-supplementary.pdf]

Supplementary file S1:

Jet Care Hydro ingredients:

Water, Phenoxyethanol, Sodium Chloride, Citric Acid, Sodium Hydroxide, Chlorphenesin, Ethylhexylglycerin, Butylene Glycol, Aloe Barbadensis Leaf Powder, Hyaluronic Acid, Silanetriol, Sorbic Acid.

Mandelic & Salicyl solution ingredients:

Water, Sodium Hydroxide, Propanediol, Mandelic Acid, Vaccinium Myrtillus Fruit Extract, Saccharum Officinarum Extract, Betaine, Sodium Polyacrylate, Malic Acid, Salicylic Acid, PPG-26-Buteth-26, PEG-40 Hydrogenated Castor Oil, Phenoxyethanol, Glycerin, Anthemis Nobilis Flower Extract, Glycolic Acid, Citrus Aurantium Dulcis Fruit Extract, Citrus Limon Fruit Extract, Lactic Acid, Allantoin, Panthenol, Acer Saccharum Extract, Chlorphenesin, PEG-12 Dimethicone, Ethylhexylglycerin, EDTA, Aloe Barbadensis Leaf Powder, Parfum, Potassium Sorbate, Citric Acid, Sodium Benzoate.

Soothing solution ingredients:

Water, Potassium Azeloyl Diglycinate, Phenoxyethanol, Glycerin, Niacinamide, Sodium Hydroxide, Aloe Barbadensis Leaf Powder, Citric Acid, Chlorphenesin, Troxerutin, Ethylhexylglycerin, Disodium EDTA, Sodium Hyaluronate, Eriophorum Spissum Flower/Stem Extract, Potassium Sorbate, Sorbic Acid.

Glow booster ingredients:

Water, Propanediol, Sodium Ascorbyl Phosphate, Saccharide Isomerate, Niacinamide, Vaccinium Myrtillus Fruit Extract, Phenoxyethanol, Sodium Hydroxide, Saccharum Officinarum Extract, Allantoin, PPG-26-Buteth-26, PEG-40 Hydrogenated Castor Oil, Citrus Aurantium Dulcis Fruit Extract, Chlorphenesin, Citrus Limon Fruit Extract, Ethylhexylglycerin, EDTA, Acer Saccharum Extract, Parfum, Sodium Citrate, Citric Acid, Linalool.

Supplementary file S2:

What Does It Measure?

The Mexameter® MX 18 is a quick, easy and **economical** tool to measure the two components mainly responsible for the colour of skin: **melanin and haemoglobin (erythema)**.

The Measuring Principle

The measurement is based on **absorption/reflection**.

The Mexameter® MX 18 probe emits 3 specific light wavelengths. A receiver measures the reflection from the skin. As the quantity of emitted light is defined, the quantity of light absorbed by the skin can be calculated.

Fields of Application

There are countless fields of application where changes in the skin colour are of interest.

- Many international scientific studies demonstrate its benefits in important skin related and **cosmetological application** fields.
- Indispensable in **efficacy testing and claim support** for cosmetics and other products (especially sunscreen, skin whitening and skin soothing products).
- In **occupational health** the skin irritation (erythema) is of special interest to educate the necessity of protection schemes.
- Other products, e.g. **foodstuffs** can also be tested.

Advantages

- The high quality electronics of the probe allow a **very quick** measurement.
- It is very sensitive to the **slightest changes** in skin colour.
- A spring in the measuring head provides a very low **constant pressure** on the skin.
- The **convenient handling** of the probe permits measurements at all body sites.
- Available for C+K **MPA-systems**, as stand-alone device (**MDD**) and **wireless probe** (operation with RR 200 & MPA WLplus software).

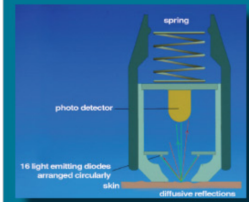

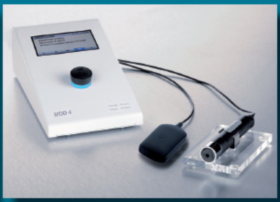

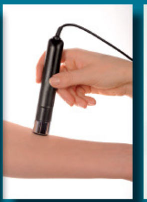

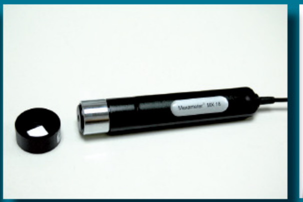

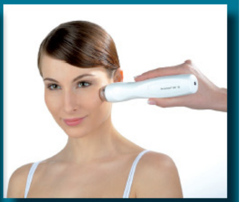

Technical Data (for probe with cable)
Dimensions: 13 cm x Ø 2.4 cm; Measuring surface: Ø 5 mm ≈ 19.6 mm²; Probe cable: 1.3 m ; Weight: 85 g incl. cable
Measurement principle: reflection
Nominal wavelengths (peaks): green: 568 nm, red: 660 nm, infrared: 880 nm
Units: arbitrary Mexameter® units (0-999), Measurement uncertainty: ± 5%
Technical changes may be made without prior notice.

Courage+Khazaka electronic GmbH since 1986
Mathias-Brüggen-Str. 91 · 50829 Köln · GERMANY
phone +49 221 95 64 99 0 · fax +49 221 95 64 99 1
info@courage-khazaka.de · www.courage-khazaka.de

2024-09
